# Enteric Protozoan Parasitosis and Associated Factors among Patients with and without Diabetes Mellitus in a Teaching Hospital in Ghana

**DOI:** 10.1155/2023/5569262

**Published:** 2023-12-09

**Authors:** Eric Konadu, Mainprice Akuoko Essuman, Angela Amponsah, Wisdom Xoese Kwadzo Agroh, Ernest Badu-Boateng, Stephen Yao Gbedema, Yaw Duah Boakye

**Affiliations:** ^1^Department of Pharmaceutics, Faculty of Pharmacy and Pharmaceutical Sciences, College of Health Sciences, Kwame Nkrumah University of Science and Technology, Kumasi, Ghana; ^2^Department of Microbiology, Parasitology Laboratory Unit, Komfo Anokye Teaching Hospital, Kumasi, Ghana; ^3^Department of Medical Laboratory Science, School of Allied Health Sciences, College of Health and Allied Sciences, University of Cape Coast, Cape Coast, Ghana; ^4^Department of Biological Sciences, Southern Illinois University Edwardsville, Edwardsville, Illinois, USA

## Abstract

**Background:**

Enteric protozoa infections (EPIs) could worsen clinical outcomes in patients with diabetes mellitus and therefore requires prompt and accurate diagnosis and attention. This study aimed to determine the burden of EPIs and their associated factors among patients with and without diabetics at the Komfo Anokye Teaching Hospital (KATH) in Ghana. Again, the diagnostic performance of parasitological techniques routinely used for diagnosis was assessed.

**Methods:**

A total of 240 participants (made up of 140 patients with diabetes and 100 patients without diabetes) were recruited into the study by simple random sampling from November 2020 to May 2021. Stool samples of participants were collected, along with their demographic information, and examined using the saline direct wet mount (DWM), formol-ether concentration (FEC), and modified Ziehl–Neelsen staining (ZNS) techniques for the presence of enteric protozoans.

**Results:**

Enteric protozoa were found among 62/140 (44.3%) diabetic patients and 13/100 (13.0%) nondiabetic patients. The predominant protozoa identified were *Cryptosporidium* spp. (17.86%) among patients with diabetes and *Blastocystis hominis* (7.0%) among patients without diabetes. EPI was associated with diabetes mellitus status (AOR = 3.48, 95% CI, 1.55–7.79), having diabetes for more than five years (AOR = 3.83, 95% CI, 1.65–8.86) and having comorbidity (AOR = 2.93, 95% CI, 1.33–6.45). The FEC technique had the highest sensitivity (100.0%), specificity 94.3% (95% CI, 91.35–97.22), and accuracy 95.0% (95% CI, 88.54–98.13) when compared to other techniques for diagnosis.

**Conclusion:**

EPIs are a significant health problem among patients with diabetes at KATH, and therefore antiparasitic drugs should be included in their treatment protocols for better health outcomes. Again, the FEC technique has demonstrated better performance in detecting EPIs and is therefore recommended to achieve early and accurate diagnosis of EPIs.

## 1. Background

Noncommunicable diseases (NCDs) remain a significant issue in healthcare and are reported to contribute to about 71% of the total number of global deaths annually according to a recent review [1]. The top four NCDs that cause the most deaths are cardiovascular diseases which causes 17.9 million deaths, cancers which causes 9.0 million deaths, respiratory diseases which causes 3.9 million deaths, and diabetes mellitus (DM) which causes 1.5 million deaths [2]. Globally, there is an increase in DM with 463 million cases reported and is projected to rise to 578 million by 2030 [3] because of a sedentary lifestyle and changing dietary patterns. Over the past decade, DM prevalence has risen faster in low- and middle-income countries compared to developed countries [3]. In Sub-Saharan Africa, 4.0% of the population is affected by DM and this number is projected to reach 24 million in the next 20 years [4]. Recent surveys in Ghana have reported DM prevalence between 5.4% and 8.5% [5–7] with the International Diabetes Federation reporting a prevalence of 2.0% among the adult population [3].

Hyperglycemia in DM can result in the suppression of both innate and adaptive immunity of patients making them immunocompromised [2]. This occurs as a result of decreased production of cytokine, inhibition of leukocyte recruitment, inability to recognize pathogens, and reduced recruitment and dysfunction of macrophage, neutrophil, complement system and natural killer cells [8]. A study conducted by Martinez et al. [9] found that there was inhibition of leukocyte recruitment in mice induced with diabetes after tracheal instillation of *Klebsiella pneumoniae*. The study further revealed that the diabetic mice recruited fewer macrophages to the alveolar space (site of infection). Another study by Berrou et al. [10] showed that in diabetics, there is ineffective degranulation of neutrophil and natural killer cells resulting in the inability of the immune system to kill invading pathogens leading to immunosuppression. The negative effect of this immunosuppression is the occurrence of opportunistic infections including intestinal parasitic infections [11–14].

The identification of enteric protozoan infections (EPIs) among immunocompromised patients including those with diabetes has become an issue of public interest [15]. EPIs in humans are associated with the immunological status of the individuals, poverty, poor environmental and personal hygiene, unsafe water supply sources, and contamination of food and water with human and animal excreta among others [16]. Enteric protozoa can colonize and dwell in the small intestine of the susceptible host and derive their nutritional requirements from the intestinal walls [17]. Infection from protozoans such as *Giardia* and *Entamoeba* causes severe chronic diarrhoea which is characterised by persistent vomiting and can also result in electrolyte (such as sodium, potassium, chloride, phosphate, and magnesium) imbalance [2, 18, 19]. EPIs also expose infected individuals to other gastrointestinal disorders such as bloating, stomach pain, and tenderness [14]. Intestinal parasitic infections could also increase the invasiveness of other microbial infections [20]. It is therefore important to ensure the early and accurate diagnosis of protozoan parasites among diabetic patients for prompt treatment and stoppage of its deleterious effects. Unfortunately, little information exists in Ghana concerning the prevalence of protozoan infections among diabetic patients and their associated risk factors.

Parasitic infections have led to a vast number of diseases from relatively innocuous to life-threatening complications which constitute a major public health concern around the globe. Approximately, 30% of the world's population is at risk of enteric parasitic infections especially immunocompromised individuals and the incidence has rapidly increased in recent years [21]. Additionally, there has been a misconception that parasitic infections occur only in the tropical areas; however, it has also gained momentum in the temperate and subtropical regions, and hence it requires urgent attention and scrutiny for better health outcomes [22].

Stool examination for enteric protozoa is crucial in making decisive clinical decisions on patients for any possible infestation and management [23]. In Ghana, the widely adopted method for routine stool examination for enteric protozoa is the direct wet mount method and only a few of the clinical laboratories use the formol-ether concentration method and modified Ziehl–Neelsen technique for routine stool examination [24]. Unfortunately, the diagnostic performance of these parasitological stool examination techniques has not been thoroughly evaluated. This study, therefore, aimed to determine the burden of EPI among patients with diabetes compared to those without diabetes and further evaluated the diagnostic performance of the stool examination methods used. The findings of this study would be helpful in initiating prompt and accurate diagnosis of EPIs among patients with diabetes in Ghana and other places.

## 2. Methods

### 2.1. Ethical Consideration

The study protocol was approved by the Ethical Review Committee of Komfo Anokye Teaching Hospital (RD/CR20/158). Participants provided written informed consent (S1) after the aim and objectives of the study were explained to them. Participants' information and laboratory results remained protected by ensuring that their names never appeared on any part of this report. Participants identified with EPIs were referred to clinicians for appropriate treatment. All methods and procedures were performed in accordance with the relevant guidelines and regulations.

### 2.2. Study Design and Setting

This was a hospital-based case-control study conducted at the Komfo Anokye Teaching Hospital (KATH) in Ghana from November 2020 to May 2021. KATH is a 1200-bed tertiary hospital and the second-largest health facility in Ghana located in Kumasi, the regional capital of the Ashanti Region. Kumasi represents 19.4% of Ghana's total population with a projected population of 4,780,380 and is located between latitudes 6.35° N and 6.40° N and longitudes 1.3° W and 1.35° W. KATH is a referral centre for 11 out of the 16 administrative regions in Ghana and is accessed by patients from neighbouring countries such as Ivory Coast and Burkina Faso. The hospital has a diabetes clinic which cares for approximately 500 patients monthly.

### 2.3. Sample Size Determination and Study Participants

The StatCalc function of the Epi-Info software version 7.2 (CDC, Atlanta, GA, USA) was used to determine the minimum sample size required for the study. Sample size calculation was performed considering a 95% confidence interval (CI), 80% power, ratio of 2 : 3 between cases and controls, and prevalence of parasitic infection of 27.5% for cases and 12.5% for controls based on an earlier study [25]. A minimum sample size of 137 cases and 96 controls (a total of 233 based on the Fleiss method for calculating sample size for case-control in the Epi-Info software) was determined to be necessary for the study.

For this study, the participants comprise 140 known diabetic patients (individuals diagnosed with diabetes and attending the diabetes clinic for monitoring and treatment) and 100 nondiabetic patients reporting at the parasitology laboratory for routine stool examination. Participants who consented to participate in the study were conveniently included in the study. Participants in the study had to meet the following requirements: Patients with type 2 diabetes mellitus who had received a clinical diagnosis and have had it for more than six months were chosen as cases, whereas controls were nondiabetics with fasting blood glucose (FBG) levels less than 6.4 mmol/L. Individuals who did not consent to participate in the study and those who had immunosuppressive disorders, pregnant or were taking immunosuppressant drugs were excluded.

### 2.4. Data Collection

#### 2.4.1. Sociodemographic Data

Participants were interviewed using a structured questionnaire (S2) to obtain information on their age, sex, educational level, occupation, and presence of comorbidities. Participants were asked to indicate their awareness of deworming and whether they had been dewormed in the last three months before the study. For diabetic participants, the number of years for which they have been diagnosed by a clinician for being diabetic was inquired.

#### 2.4.2. Stool Sample Collection

The participants were given clean, dry, leak-proof, and wide-mouthed plastic stool containers to provide stool specimens after the interview. They were educated on preanalytical errors as a precautionary measure to avoid contamination of the stool specimen. Participants who could not produce the stool samples on the spot at the hospital premises were asked to go home with the specimen containers and bring fresh passed stool samples the following day. Stool specimen collected from the participants was appropriately labelled and transported to the parasitology laboratory of the same hospital within an hour for examination.

### 2.5. Laboratory Procedures

#### 2.5.1. Macroscopic Examination of the Stool Sample

Stool samples were first examined macroscopically for physical characteristics such as consistency and colour.

#### 2.5.2. Microscopic Examination of the Stool Sample

The parasitological methods used in the identification of enteric protozoa included the saline direct wet mount technique with Lugol's iodine, the formol-ether concentration technique with Lugol's iodine, and the Modified Ziehl–Neelsen staining technique for intestinal protozoans.

*(1) Saline Direct-Wet Mount Technique*. Stool samples were examined using saline direct wet mount techniques as described in a previous study [14]. Approximately 2 g of the freshly collected stool samples were placed in a clean leak-proof 40 mL plastic stool container. About ten millilitres of normal saline (0.9% w/v) was added to the stool specimen and emulsified using an applicator stick. Pasteur's pipette was used to fetch about 500 microliters of the emulsified stool specimen onto two different clean grease-free 20 mm × 20 mm slides. To both of the slides, Lugol's iodine was added and mixed gently. The same process was repeated for the second slide for a comprehensive examination of each specimen. The slides were then covered with coverslips and examined under a microscope with ×10 and ×40 objectives lens for motile trophozoites and cysts of enteric protozoa.

*(2) Formol-Ether Concentration Technique*. The formol-ether concentration technique as used in a previous study was employed [14]. About 9 mL of the remaining stool specimen from the direct wet mount technique was filtered through a double-layered gauze into a 50 mL beaker. Four millilitres of 10% formalin was added to the filtrate, mixed thoroughly, and allowed to stand at room temperature for 10 minutes to achieve adequate fixation. The resulting mixtures were then transferred into 10 mL centrifuge tubes to the 7 mL mark after which 3 mL diethyl ether was added. The content in the tubes was mixed, capped, and centrifuged at 3000 rpm for 3 minutes. The supernatant containing diethyl ether dissolved faecal debris and formol-saline was discarded. The sediment at the bottom of the tube containing the enteric protozoa was resuspended using 0.9% NaCl (normal saline). Using Pasteur's pipette, two drops of the resuspended sediment were placed on two separate clean labelled grease-free glass slides. A drop of Lugol's iodine stain was added to one of the slides and covered with a coverslip after gentle mixing. The slides were examined for cysts and trophozoites of enteric protozoa using ×10 and ×40 objective lenses.

*(3) Modified Ziehl–Neelsen Technique*. The modified Ziehl–Neelsen staining technique as used in a previous study by Sisu et al. [14] was adopted with slight modification. From the remaining stool sample of the resuspended sediment from the formol-ether concentration technique, a faecal smear was made and air-dried. It was then fixed in methanol for 3 minutes and stained with Carbol fuschin for 10 minutes. The stained slide was rinsed thoroughly under tap water and decolourized in acid alcohol (1% HCL in methanol) for 15 seconds. It was then counterstained with methylene blue for 60 seconds, rinsed again thoroughly under tap water, and air-dried. The dried slide was examined using an ×100 oil immersion objective lens for enteric protozoans.

### 2.6. Quality Control

All the reagents were checked for contamination each time they were used. For all the parasitological methods used in the study, a preserved stool specimen which is positive for enteric protozoa was included in each batch of analysis for all the methods. Stool analyses were conducted by individuals who were blinded such that they did not know whether samples were coming from diabetics or nondiabetics. Additionally, the positive and negative samples were not discarded immediately but were further confirmed by a supervising clinical parasitologist before reporting.

### 2.7. Statistical Analysis

All results were entered into Microsoft Excel 2016 and analysed using the IBM SPSS version 26.0 statistical software (IBM Corporation, Armonk, State of New York). The demographics and clinical data, as well as the results of the parasitological examination of participants, were analysed using descriptive statistics. The categorical characteristics of the study participants were compared using the Chi-square test. Continuous variables such as age were reported using means and standard deviations. To identify the association between the demographic variables of participants and the risk of EPI, multivariate logistic regression was performed on all variables with *P* values <0.25 in the univariate logistic regression. The results of the logistic regression analysis were reported using odds ratios with their corresponding 95% confidence intervals. All analyses were two-sided with *P* values <0.05 considered statistically significant.

Sensitivity, specificity, positive predictive value (PPV), negative predictive value (NPV), and accuracy were used to compare the diagnostic techniques employed in the study [26]. Sensitivity, specificity, PPV, NPV, and accuracy were defined as follows: sensitivity = number of true positives/(number of true positives + number of false negatives); specificity = number of true negatives/(number of false positives + number of true negatives); PPV = number of true positives/(number of true positives + number of false positives); NPV = number of true negatives/(number of false negatives + number of true negatives); and accuracy = (number of true positives + number of true negatives)/(number of true positives + number of true negatives + number of false positives + number of false negatives). Agreement analysis between the diagnostic techniques was performed using the Kappa (*κ*) statistic using GraphPad's QuickCalcs online calculator (https://www.graphpad.com/quickcalcs/kappa1/). Altman's benchmark scale was used to measure the strength of agreement according to the *κ* value, with the scores divided into <0.20 poor; 0.21–0.40 fair; 0.41–0.60 moderate; 0.61–0.80 good; 0.81–1.00 very good. Summary measures were expressed as means and 95% confidence intervals (CIs).

## 3. Results

### 3.1. Sociodemographic and Clinical Characteristics of Study Participants

Table 1 summarises the characteristics of the study participants. In this study, 240 participants made up of 140 diabetic patients and 100 non-diabetic patients were enrolled. The average age of participants was 56.73 + 12.11 with the youngest being 35 years and the oldest being 85 years old. The larger portion of participants were females (78.8%), had primary-level education (35.8%), and were employed (64.2%). More than half of the participants (65.8%) indicated that they were aware of deworming. However, only 42.9% of them had dewormed in the last three months before the study. Diabetic patients recruited for the study differed significantly from nondiabetic patients in terms of age, gender, educational status, employment status, and comorbidity. Diabetic patients were significantly older than nondiabetic patients (60.79 + 10.47 vs. 51.03 + 12.01). Most of the diabetic patients had primary education 63 (45.0%) whereas among the nondiabetic patients, most of them had no formal education 30 (30.0%). In terms of employment, 55.0% of diabetic patients were employed while 77.0% of non-diabetic patients were employed. Comorbidity was found to be more predominant among diabetic patients (75.0%) than nondiabetic patients (17.0%).

### 3.2. Enteric Protozoan Infection among Participants

**Figure 1:**
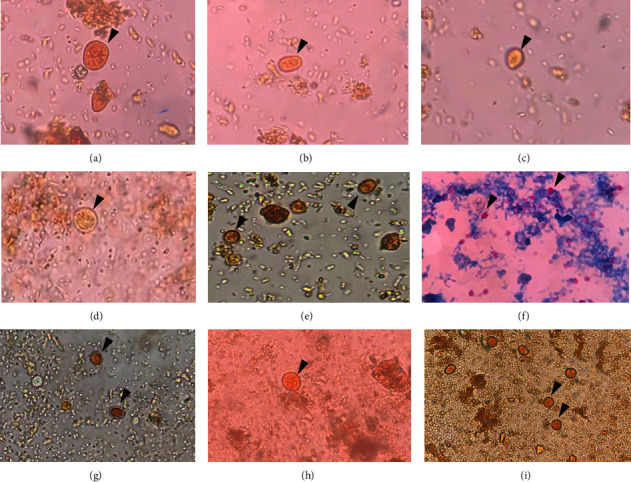
Photomicrographs of some intestinal protozoans recovered during the study. (a, i) *Entamoeba histolytica/dispar cyst* (FEC iodine stain, ×400), (b, e, g) *Giardia lamblia cyst* (FEC iodine stain, ×400), (c) *Chilomastix mesnili cyst* (FEC iodine stain, ×400), (d) *Blastocystis hominis cyst* (FEC iodine stain, ×400), (f) *Cryptosporidium spp. oocyst* (MZN stain, ×100), and (h) *Entamoeba coli cyst.*FEC–formol-ether concentration technique. MZN: modified Ziehl–Neelsen staining technique.

In all, 62/140 (44.3%) of diabetic patients and 13/100 (13.0%) of nondiabetic patients harboured one or more enteric protozoan parasites (Table 2). Seven different enteric protozoan parasite species were identified among the study participants. The predominant protozoa isolated among diabetic patients was *Cryptosporidium* spp. 25 (17.86%) while *Blastocystis hominis* 7 (7.0%) was the most predominant among nondiabetic patients. Cryptosporidiosis was significantly higher among patients with diabetes 25 (17.86%) compared to those without diabetes 1 (1.00%). Double and triple coinfection was observed among twenty-two diabetic patients and one nondiabetic patient. *Cryptosporidium* spp. and *Cyclospora cayetanensis* coinfection were the most predominant among diabetic patients 6 (4.29%). Among nondiabetic patients, *B. hominis* + *E. nana* + *Cryptosporidium* spp. was the only coinfection observed.  Figure 1 shows micrographs of some of the isolated protozoan parasites in participants' stool specimens.

### 3.3. Factors Associated with Enteric Protozoan Parasitic Infections

Logistic regression analysis was used to assess the factors associated with EPIs among the studied population (Table 3). Univariate analysis showed that infection with any protozoan parasite was significantly associated with diabetes status, years with diabetes, and the presence of comorbidity. Multivariate analysis showed that a patients with diabetes were 3.48 times more likely (AOR = 3.48, 95% CI = 1.55–7.79) to have EPI compared to nondiabetic patients. Additionally, an increased duration of diabetes for more than 5 years was associated with an increased odds of EPI (AOR = 3.83, 95% CI, 1.65–8.86). Likewise, participants with comorbidity were more likely to harbour protozoan parasites (AOR = 2.93 95% CI, 1.33–6.45) than those without comorbidities.

### 3.4. Comparison of Diagnostic Techniques for the Diagnosis of Enteric Parasitosis

Table 4 shows the number of participants in whom the various diagnostic techniques were able to detect protozoan parasites. The DWM technique detected enteric protozoans among 30 (12.5%) of the participants while the FEC and MZN detected enteric protozoans among 42 (17.5%) and 46 (19.2%) of participants, respectively. Sensitivity, specificity, PPV, NPV, and accuracy were determined for the FEC and MZN staining using direct wet mount as the gold standard (Table 5). The FEC technique had higher sensitivity (100.0%), specificity (94.3%), and accuracy (95.0%). The PPV and NPV were also higher for FEC. The MZN staining technique had low sensitivity (33.3%) but had appreciable specificity 82.9% and accuracy (76.7%).

## 4. Discussion

Diabetes mellitus is a metabolic disorder that causes numerous morbidities and mortalities globally [27]. Due to the immunosuppressive nature of the condition, it subjects the affected individuals to easy invasion and colonization by enteric protozoa which can result in life-threatening complications and poor prognosis [28]. This makes the geospatial estimation of the burden of enteric protozoa parasitosis and its associated risk factors among diabetics extremely important. Also, evaluating the diagnostic performance of various methods used in the detection of enteric protozoa is crucial to ensure an accurate diagnosis by recommending the best method.

In the present study, the overall prevalence of EPIs among diabetic patients was 82.67% while 17.33% was recorded among nondiabetic patients. This finding is consistent with similar studies [12, 22, 29–31] that reported higher infection of EPIs among patients with diabetes compared to nondiabetic patients. However, the study disagrees with an earlier study in Cameroon [32] which reported a higher prevalence among nondiabetic patients at 23.5% compared to diabetic patients at 10.0%. The difference in prevalence could be linked to the different geographic settings where the study took place, the methods used in the analysis of the stool samples, and endemicity of EPIs in the study area. The high prevalence of EPIs among diabetics could be due to perpetual inflammation which results in reduced cytokine production and defects in phagocytic activities by macrophages and polymorphonuclear neutrophils [8]. This eventually leads to an inability of the immune system to effectively handle invading pathogens including enteric protozoa, hence the high prevalence of EPIs among patients with diabetes.

The study found that *Cryptosporidium* spp. was the most prevalent single intestinal protozoa detected, and the highest biparasitic enteric protozoa infection recorded was *Cryptosporidium* spp. *+* *Cyclospora cayetanensis*. A total of five triple enteric protozoa infections were also reported among the study participants (Table 2). In all the instances, the patients with diabetes were infected with the individual enteric protozoa compared to nondiabetic patients. These present findings are in line with similar studies conducted among persons with diabetes in Southern Ethiopia [33] and Egypt [34] which recorded a higher prevalence of *Cryptosporidium* spp. *Cryptosporidium* spp. is an opportunist intestinal coccidian and causes detrimental effects among immunosuppressed individuals including those with diabetes, HIV, and cancer [35]. Therefore, the immunosuppresed state of patients with diabetes makes *Cryptosporidium* spp. capitalizes on the porosity of the immune system to exhibit its pathogenicity. The nature of parasites isolated from the studied population may be influenced by food consumption practices, water treatment practices, and hygienic conditions.

The study further reports that patients with diabetes had 3.48 times higher odds of being infected with enteric protozoa parasites. This agrees with a similar study which found a higher odd of EPIs among diabetic patients [30]. This could be related to their low immunological status and their susceptibility to secondary infections. Also, participants who have had diabetes for more than five years had 3.83 times higher odds of contracting enteric protozoa infections. Some similar studies have earlier reported an association between the duration of DM and the aquisition of enteric parasitic infection [14, 30]. This is contrary to other studies which reported no association between the duration of DM and the acquisition of EPIs [12, 36]. This might be due to environmental factors and the differences in the quality of healthcare that exist in the study sites and their influence on controlling secondary infections in patients with diabetes. The study further showed that having comorbidities makes the individual 2.93 times more vulnerable to getting infected with EPIs. This is because multiple medical conditions further deteriorate immunity against infections making the body susceptible to EPIs [37].

The current study evaluated the diagnostic performance of the parasitological examination methods used in the study using the direct wet mount technique as the gold standard. This is because the direct wet mount technique is the widely used and adopted parasitological method in the study setting. We found that formol-ether concentration had a better sensitivity, specificity, and accuracy compared to the modified Ziehl–Neelsen staining method in the detection of EPIs and compared well with the gold standard with a kappa value of 0.805. The high sensitivity and specificity of the formol-ether concentration technique in detecting enteric protozoa could be due to the holistic preparation process the method employs and the unique role each reagent plays in getting concentrated sediment for examination. Before the filtration process, 10% formalin is used as a fixative that preserves the integrity of cysts and trophozoites that might be present in the specimen. The diethyl ether dissolves debris and the centrifugation process concentrates and sediments the enteric parasites for examination [38]. For diagnostics and detection rate, the modified Ziehl–Neelsen staining method was able to detect more of the intestinal coccidian compared to formol-ether concentration targeting both the intestinal coccidian and other enteric protozoa and the least performed is the direct wet mount technique. It may therefore be advantageous to use different parasitological examination techniques for better diagnosis of EPIs.

The findings of this study should be considered taking into consideration these limitations: The first was the inability to estimate the more sensitive glycated haemoglobin of diabetic patients to confirm the FBG results as a glycaemic control indicator. Second, our inability to use Kato-Katz, molecular, or immunofluorescence techniques may have decreased the likelihood of detecting some parasites in the samples of patients. This study was conducted in a single setting for which reason the findings may not represent the situation at other facilities. Finally, the addition of participants with comorbidities and those on anthelminthics presents a limitation for the study as these could impact parasitic infection.

## 5. Conclusions

The prevalence and distribution of the enteric protozoa parasites were higher among patients with diabetes compared to those without diabetes. *Cryptosporidium* spp. was the highest encountered enteric protozoa, and most of the infestation was recorded among the patients with diabetes. The predominant polyparasitism detected was *Cryptosporidium* spp. *+* *Cyclospora Cayetanensis* which was also higher among the diabetic patients compared to nondiabetic patients. Having diabetes, particularly for a longer time as well as having comorbidities is strongly associated with enteric protozoa infections. Formol-ether concentration technique exhibited high sensitivity and specificity compared to ZN staining in the detection of enteric protozoans. It is recommended that patients with diabetes should routinely be screened for enteric protozoa and treated when infected. It is also recommended that different stool examination methods should be used in the diagnosis of enteric protozoa and not solely rely on the direct wet mount technique as is widely seen in clinical laboratories in Ghana.

## Figures and Tables

**Table 1:** Sociodemographic and clinical characteristics of study participants.

Characteristics	Total	Diabetics	Nondiabetics	*X* ^2^	*p* value
Age	56.73 + 12.11	60.79 + 10.47	51.03 + 12.01	5.74	<0.001
Age groups					
<60	132 (55.0)	60 (42.9)	72 (72.0)	20.01	<0.001
60+	108 (45.0)	80 (57.1)	28 (28.0)		
Gender					
Female	189 (78.8)	116 (82.9)	73 (73.0)	3.39	0.047
Male	51 (21.3)	24 (17.1)	27 (27.0)		
Educational status					
No formal education	65 (27.1)	35 (25.0)	30 (30.0)	14.37	0.002
Primary	86 (35.8)	63 (45.0)	23 (23.0)		
Secondary	31 (12.9)	12 (8.6)	19 (19.0)		
Tertiary	58 (24.2)	30 (21.4)	28 (28.0)		
Employment status					
Not employed	86 (35.8)	63 (45.0)	23 (23.0)	12.28	<0.001
Employed	154 (64.2)	77 (55.0)	77 (77.0)		
Years with condition					
<1	5 (2.1)	5 (3.6)	—		
2 to 5	12 (5.0)	12 (8.6)	—		
>5	123 (51.2)	123 (87.9)	—		
Awareness of deworming					
No	82 (34.2)	46 (32.9)	36 (36.0)	0.26	0.356
Yes	158 (65.8)	94 (67.1)	64 (64.0)		
Dewormed in the last 3 months					
No	137 (57.1)	74 (52.9)	63 (63.0)	2.45	0.076
Yes	103 (42.90)	66 (47.1)	37 (37.0)		
Has comorbidity					
No	118 (49.2)	35 (25.0)	83 (83.0)	75.52	<0.001
Yes	122 (50.8)	105 (75.0)	17 (17.0)		

**Table 2:** Distribution of enteric protozoa among patients with and without diabetes.

Detected parasites	Total (*n* = 240)	Diabetics (*n* = 140)	Nondiabetics (*n* = 100)	*p* value
*Single infection (n* *=* *28)*				
*Blastocystis hominis*	13 (5.42)	6 (4.29)	7 (7.0)	0.360
*Entamoeba coli*	5 (2.08)	3 (2.14)	2 (2.00)	0.939
*Chilomastix mesnili*	1 (0.42)	1 (0.71)	0 (0.00)	0.397
*Entamoeba histolytica/dispar*	3 (1.25)	2 (1.43)	1 (1.00)	0.768
*Giardia lamblia*	3 (1.25)	2 (1.43)	1 (1.00)	0.768
*Cryptosporidium* spp.	26 (10.83)	25 (17.86)	1 (1.00)	<0.001
*Cystoisospora belli*	1 (0.42)	1 (0.71)	0 (0.00)	0.397

*Double infections (n* *=* *18)*				
*B. hominis* + *Cryptosporidium* spp.	2 (0.83)	2 (1.43)	0 (0.00)	0.230
*B. hominis* + *E. histolytica/dispar*	1 (0.42)	1 (0.71)	0 (0.00)	0.397
*Cryptosporidium* spp. *+* *C. cayetanensis*	6 (2.50)	6 (4.29)	0 (0.00)	0.036
*G. lamblia* + *Cryptosporidium* spp.	3 (1.25)	3 (2.14)	0 (0.00)	0.141
*G. lamblia* + *C. cayetanensis*	1 (0.42)	1 (0.71)	0 (0.00)	0.397
*E. coli* + *G. lamblia*	1 (0.42)	1 (0.71)	0 (0.00)	0.397
*E. nana* + *Cryptosporidium* spp.	1 (0.42)	1 (0.71)	0 (0.00)	0.397
*E. histolytica/dispar* + *Cryptosporidium* spp.	2 (0.83)	2 (1.43)	0 (0.00)	0.230
*E. histolytica/dispar* + *G. lamblia*	1 (0.42)	1 (0.71)	0 (0.00)	0.397

*Triple infection (n* *=* *5)*				
*E. histolytica/dispar* + *G. lamblia* + *Cryptosporidium* spp.	1 (0.42)	1 (0.71)	0 (0.00)	0.397
*E. coli* + *G. lamblia* + *Cryptosporidium* spp.	1 (0.42)	1 (0.71)	0 (0.00)	0.397
*B. hominis* + *E. nana* + *Cryptosporidium* spp.	1 (0.42)	0 (0.00)	1 (1.00)	0.236
*B. hominis* + *C. mesnili* + *E. histolytica/dispar*	1 (0.42)	1 (0.71)	0 (0.00)	0.397
*B. hominis* + *E. coli* + *Cryptosporidium* spp.	1 (0.42)	1 (0.71)	0 (0.00)	0.397

**Table 3:** Risk factors of intestinal protozoa infections among the study participants.

Characteristics	Enteric protozoan parasites		COR (95% CI)	*p* value	AOR (95% CI)	*p* value
	Negative (*n* = 165)	Positive (*n* = 75)				
*Age group*						
<60	96 (58.18)	36 (48.00)	1		1	
60+	69 (41.82)	39 (52.00)	1.51 (0.87–2.61)	0.143	0.68 (0.24–1.93)	0.467

*Gender*						
Female	127 (76.97)	62 (82.67)	1		—	—
Male	38 (23.03)	13 (17.33)	0.70 (0.35–1.41)	0.319	—	—

*Educational status*						
No formal education	41 (24.85)	24 (32.00)	1		1	
Primary	55 (33.33)	31 (41.33)	0.96 (0.49–1.88)	0.912	0.78 (0.37–1.67)	0.528
Secondary	25 (15.15)	6 (8.00)	0.41 (0.15–1.14)	0.088	0.54 (0.17–1.73)	0.296
Tertiary	44 (26.67)	14 (18.67)	0.54 (0.25–1.19)	0.128	0.64 (0.26–1.56)	0.321

*Employment status*						
Not employed	59 (35.76)	27 (36.00)	1		—	—
Employed	106 (64.24)	48 (64.00)	0.99 (0.56–1.75)	0.971	—	—

*Diabetes status*						
Nondiabetics	87 (52.73)	13 (17.33)	1		1	
Diabetics	78 (47.27)	62 (82.67)	5.32 (2.72–10.41)	<0.001	3.48 (1.55–7.79)	0.002

*Years with condition*						
0	87 (52.73)	13 (17.33)	1		1	
1	4 (2.42)	1 (1.33)	1.67 (0.17–16.15)	0.656	1.46 (0.14–15.36)	0.751
2 to 5	8 (4.85)	4 (5.33)	3.35 (0.88–12.71)	0.076	2.67 (0.64–11.18)	0.179
>5	66 (40.0)	57 (76.00)	5.78 (2.92–11.43)	<0.001	3.83 (1.65–8.86)	0.002

*Has comorbidity*						
No	99 (60.00)	19 (25.33)	1		1	
Yes	66 (40.00)	56 (74.67)	4.42 (2.41–8.11)	<0.001	2.93 (1.33–6.45)	0.008

*Awareness of deworming*						
No	51 (30.91)	31 (41.33)	1		1	
Yes	114 (69.09)	44 (58.67)	0.64 (0.36–1.12)	0.116	0.53 (0.27–1.04)	0.066

*Dewormed in the last 3 months*						
No	97 (58.79)	40 (53.33)	1		—	—
Yes	68 (41.21)	35 (46.67)	1.25 (0.72–2.16)	0.429	—	—

COR: crude odds ratio; AOR: adjusted odds ratio; CI: confidence interval; 1: reference.

**Table 4:** Recovery rate of the different parasitic examination methods.

Diagnostic technique	No. of parasites detected	Percentage (%)
Direct wet mount with and without Lugol's iodine	30	12.5
Formol-ether concentration with Lugol's iodine	42	17.5
Modified Ziehl–Neelsen staining	46	19.2

**Table 5:** Agreement between the different techniques in detecting protozoan parasites using the wet mount preparation technique as a reference.

Diagnostic test		Direct wet mount		% Sensitivity (95% CI)	% Specificity (95% CI)	% PPV (95% CI)	% NPV (95% CI)	% Accuracy (95% CI)	Kappa index (95% CI)
		−	+						
		*n* (%)	*n* (%)						
Formol-ether concentration	−	198 (94.29)	0 (0.00)	100.00	94.29 (91.35–97.22)	71.43 (65.71–77.14)	100.00	95(88.54–98.13)	0.805 (0.699–0.910)
	+	12 (5.71)	30 (100.00)						

Modified Ziehl–Neelsen staining	−	174 (82.86)	20 (66.67)	33.33 (27.37–39.30)	82.86 (78.09–87.63)	21.74 (16.52–26.96)	89.69 (85.84–93.54)	76.7 (67.79–84.22)	0.132 (−0.012–0.275)
	+	36 (17.14)	10 (33.33)						

## Data Availability

The datasets used during the current study are available from the corresponding author on reasonable request.

## Supplementary Materials

Supplementary Materials S1: informed consent form; S2: questionnaire for the interview.
