# Supplementary material for: Enteric Protozoan Parasitosis and Associated Factors among Patients with and without Diabetes Mellitus in a Teaching Hospital in Ghana
Source: Int J Microbiol. 2023 Dec 9;2023:5569262. doi: 10.1155/2023/5569262 (PMC10725318; doi:10.1155/2023/5569262)
Supplement: Supplementary Materials — S1: informed consent form; S2: questionnaire for the interview. [file 5569262.f1.zip › S1 - Informed consent form.pdf]

INFORMED CONSENT

Title of Research: ***Enteric protozoan parasitosis and associated factors among patients with and without diabetes mellitus in a Teaching Hospital in Ghana***

Principal Investigator: Mr. Eric Konadu

Department of Pharmaceutics, Faculty of Pharmacy and Pharmaceutical Sciences, College of Health Sciences, Kwame Nkrumah University of Science and Technology, Kumasi, Ghana.

Email: erickonadu12@gmail.com

Supervisor: Prof Yaw Duah Boakye

Department of Pharmaceutics, Faculty of Pharmacy and Pharmaceutical Sciences, College of Health Sciences, Kwame Nkrumah University of Science and Technology, Kumasi, Ghana.

Email: ydboakye.pharm@knust.edu.gh

Introduction and Purpose of the Study

People with diabetes are usually prone to intestinal parasite infections. This situation worsens the already-known complications of diabetes with an increased death rate. The study therefore seeks to determine the burden of intestinal parasite infections among patients with diabetes compared to those without diabetes. The study will further assess the routine methods used in testing intestinal parasite infections if are very effective.

Description of the Research

When you enter the program, you will be asked to complete a questionnaire. After completing the questionnaire, if you meet the selection criteria based on the questions asked will be instructed to bring a stool sample for analysis for intestinal parasites.

Subject Participation

We estimate that 240 people will be enrolled in the study. One hundred and forty (140) were people with diabetes and 100 of the participants were without diabetes. All the participants must not have any medical conditions that reduce their immunity aside from diabetes and also not take drugs that cause a reduction in immunity.

Potential Risks and Discomfort

There is absolutely no risk in participating in this study.

Potential Benefits

People who participate in the study will get to know if they have intestinal parasite infections and will be alerted for prompt treatment. Additionally, participants in this study may have a better understanding of treatment methods that will increase their general well-being.

Confidentiality

Your responses are completely anonymous. No personal identifying information will be made public, and it will be maintained throughout the study.

Authorization

I authorize the use of my data, observations, and findings found during the study period for education, presentation, and publication purposes.

Compensation

Subjects will not be compensated for participation in this study.

Voluntary Participation

Your decision to participate in the study is completely voluntary. Therefore, if you decide not to participate in the study, it will not affect the care, services, or benefits to which you are entitled.

Withdrawal from the study

If you decide to participate in the study, you may withdraw from your participation at any time without penalty.

Cost

There is no cost to participating in the study.

I voluntarily agree to participate in the study [Yes] [No]

Name of Participant………………………………………………

Date………………………………………………………………...

Name of Witness……………………..…………………………….

Date ………………………………………..……………………
