# Supplementary material for: Enteric Protozoan Parasitosis and Associated Factors among Patients with and without Diabetes Mellitus in a Teaching Hospital in Ghana
Source: Int J Microbiol. 2023 Dec 9;2023:5569262. doi: 10.1155/2023/5569262 (PMC10725318; doi:10.1155/2023/5569262)
Supplement: Supplementary Materials — S1: informed consent form; S2: questionnaire for the interview. [file 5569262.f1.zip › S2 - Questionnaire.pdf]

QUESTIONNAIRE

Enteric protozoan parasitosis and associated factors among patients with and without diabetes mellitus in a Teaching Hospital in Ghana

Demographic Characteristics

1. Identify Number…………………………….……
2. Nationality……………………….…………
3. Place of Residence…………………………......
4. Age ……………………………………...…….
5. Gender:[ ] Male [ ] Female
6. Educational background:
   a) Formal education [ ] Primary [ ] Vocational [ ] Secondary [ ] University and
   b) Non-formal education [ ]
7. Occupation…………………………..………………………………….
8. Contact / Address (***optional)***……………………………………………
9. How many years since you were diagnosed with type 2 diabetes?
   a) Less than a year [ ] b) 1-2years [ ] c) 2-5years [ ] d) more than five years [ ]
10. Do you have any knowledge of deworming?
    Yes ( ) No ( )
11. Have you been dewormed for the past three months?
    Yes ( ) No ( )
12. Are you on any immunosuppressive drug?
    Yes ( ) No ( )
13. Do you have any medical condition aside from diabetes?
    Yes ( ) No ( )
    If yes state……………………………………………
